# Something from nothing: Estimating consumption rates using propensity scores, with application to emissions reduction policies

**DOI:** 10.1371/journal.pone.0185538

**Published:** 2017-10-11

**Authors:** Nicholas Bardsley, Milena Büchs, Sylke V. Schnepf

**Affiliations:** 1 School of Agriculture, Policy and Development, University of Reading, Reading, United Kingdom; 2 Walker Institute for Climate System Research, University of Reading, Reading, United Kingdom; 3 School of Earth and Environment, University of Leeds, Leeds, United Kingdom; 4 Sustainability Research Institute, University of Leeds, Leeds, United Kingdom; 5 European Commission, Joint Research Centre, Ispra, Italy; Universitat Jaume I, SPAIN

## Abstract

Consumption surveys often record zero purchases of a good because of a short observation window. Measures of distribution are then precluded and only mean consumption rates can be inferred. We show that Propensity Score Matching can be applied to recover the distribution of consumption rates. We demonstrate the method using the UK National Travel Survey, in which c.40% of motorist households purchase no fuel. Estimated consumption rates are plausible judging by households’ annual mileages, and highly skewed. We apply the same approach to estimate CO_2_ emissions and outcomes of a carbon cap or tax. Reliance on means apparently distorts analysis of such policies because of skewness of the underlying distributions. The regressiveness of a simple tax or cap is overstated, and redistributive features of a revenue-neutral policy are understated.

## Introduction

A potential problem in survey sampling is that a phenomenon of interest occurs infrequently relative to the period in which data is collected, leading to zero-inflated data. There are many circumstances in which this can arise. For example, if a wildlife survey runs camera traps for a short time, negative results may obtain even where a target species is known to reside [1]. In the social sciences a key example is purchase infrequency. This occurs when a consumption diary is used to record buying over a relatively short duration, typically 1–2 weeks. Sampled households often record no purchase, even if they are known to consume the good in question. All households consume clothing, but many will not make purchases in a given two weeks, which is the period in the UK’s Living Costs and Food Survey for example.

Consumption surveys aim to measure rates of consumption, which unlike quantities purchased do not depend on the length of the observation window. The same drinking rate, for example, can be expressed as 1 pint per day, 7 pints per week or 365 pints per year. In a well-designed and executed survey, the estimated mean consumption rate is not biased by purchase infrequency. For zeroes will tend to be counterbalanced by positive values that, if interpreted as consumption rates, would be too high. For example, if each household used a 2l bottle of milk every 2 weeks, an ideal survey with a 1 week diary would be expected to record 50% of units with no purchase and 50% purchasing 2l. The expected mean rate of 1l per week is correct, but no entry would record 1l. The data are therefore uninformative about a given household’s consumption rate, and therefore about any other statistic than the mean. For many questions of social and political interest, there is therefore a relative paucity of information. A good example (analysed later in this paper) is CO_2_ emissions reduction policy, including carbon taxes or caps. One would like to estimate quantiles of the financial impact of such a policy, to judge its potential regressiveness and likely resistance to its implementation, for example. But key variables needed for the analysis, including consumption of flights and motor fuels, exhibit acute purchase infrequency.

A substantial literature on infrequent purchase exists in econometrics [2], with the following characteristics. Studies estimate either Engel curves or demand equations often targeting a complete system of equations for different types of good, accounting for a household’s budget [3–12]. Infrequent purchase is dealt with by specifying separate equations for the purchasing decision and for budget share, expenditure or quantity, which are estimated jointly. As more than one potential explanation exists for a given zero purchase, and information is usually not available on, for example, who actively abstains from a particular good, identifying assumptions are required for different categories of zeros. These features imply sophistication but also relatively large numbers of parameters, and rely on *a priori* model specifications. Gibson and Kim [13] test several of the infrequent purchase models, using a dataset containing both stock use and purchasing decisions, arguing that they exhibit substantial biases. Whether or not these models are inherently biased, we find it plausible that they can be mis-specified for the data to hand, so there is a need for credible specification checks.

Our aim is more limited and descriptive than such demand modelling, namely to recover the distribution of consumption rates of one good under purchase infrequency. This restricted goal enables a simpler approach. In common with many of the econometric models we estimate a purchase decision equation via a binary regression model. But the quantitative aspect is here dealt with by matching, avoiding the additional assumptions on functional form and error structure required by a second regression equation. Our proposal complements the existing methods in two ways. Firstly, one can predict values of a dependent variable using an estimated quantity regression equation and compare the values obtained to those derived by matching. This provides a model specification check. If the distributions are markedly different one might try, for example, a different functional form or error structure. Given the relative simplicity of matching, it is reasonable for the burden of achieving conformity to fall on the econometric model.

Secondly, matching is reliant on a condition of “common support” [14]. This condition is also important for an econometric model, since if it is not satisfied, counterfactual behaviour will be estimated on observations of dissimilar respondents [15]. Matching algorithms report on common support and therefore indicate whether quantitative regression involves such extrapolation. We apply Propensity Score Matching (PSM) [14] to estimate the distribution of rates of consumption. This is, to our knowledge, a novel application of PSM. Little [16] applies propensity score weighting to missing data problems in sample surveys, but does not consider purchase infrequency. The latter is not a missing data problem, since it arises even if purchase diaries are fully completed. We derive the theoretical case for using PSM to impute consumption rates in the next section. We then estimate household motor fuel consumption rates using data from the UK’s National Travel Survey (NTS) and evaluate the imputation statistically. Here we exploit favourable features of the NTS to defend against the common criticism of PSM that relevant factors are excluded from the binary regression model. We then extend the analysis to study emissions reduction policies for household motor fuels.

## Theory: using PSM to estimate consumption rates under infrequency of purchase

Let Z denote a binary event; Z = 1 if a motorist household purchased fuel and Z = 0 otherwise. Let *r* denote a potential survey outcome, the quantity of fuel purchased conditional on Z = 1. If Z = 1, *r* is recorded, as the fuel purchase value in the dataset, otherwise 0 is recorded and *r* is unknown. We first estimate the missing values of *r*. We then use *r* in conjunction with propensity scores to estimate consumption rates, *c*. For households without vehicles, *c* ≡ 0. The sampling approach here, restricting attention to vehicle owners, is identical to that in regular applications of PSM. For example, a study constructing a control group to analyse a smoking intervention will estimate the propensity score on a sample of smokers to control for self-selection into the intervention, with non-smokers falling outside the population of interest [17–19].

A propensity score, *ps*_*i*_(**X**), is the conditional probability that Z occurs, given a vector of observed characteristics **X** of a unit of observation *i*. Rosenbaum and Rubin [14] show that the *ps* is a ‘balancing score’, meaning that the distribution of **X** will tend to be the same for random samples of units with the same value of *ps*(**X**), whether Z = 1 or Z = 0. That is,

X⊥Z|ps(X) (1)

Balancing in this sense is a large sample property of *ps*. The true *ps* is always unknown and can only be estimated, for example using a binary regression model. Rosenbaum and Rubin [14] also show that *ps* can be used to correct for certain kinds of selection biases. The usual application is in the estimation of effect sizes for observational studies, to control for self-selection into a treatment group. Here in contrast we account for self-selection into the category of purchasers during a diary window. The key conditions required to estimate the sample distribution of *r* are

$$\begin{array}{l} \;r⊥Z|X\\ and\\ \;\forall X\;0<p(Z=1|X)<1 \end{array}$$ (2)

(2) is known as ‘strong ignorability’. The first part means there are no unobserved confounders, that is, unrecorded variables that affect both the probability of purchase and the quantity *r*. The second part, common support, means that there is no **X** such that Z is perfectly predictable. Given (2) it also follows that

$$\begin{array}{l} \;r⊥Z|ps(X)\\ and\\ \;\forall X\;0<p(Z=1|ps(X))<1 \end{array}$$ (3)

From (1), estimated propensity scores, $\hat{ps}(X)$, of sufficient quality can always be used to balance samples on their observed characteristics. (3) implies additionally that each household *i*: Z = 0, can be matched with a household *j*: Z = 1 with approximately the same value of $\hat{ps}(X)$ to estimate an unobserved value of *r*:

$${\hat{r}}_{i:Z_{i}=0}=r_{j:{\hat{ps}}_{j}(X)≅{\hat{ps}}_{i}(X),\;Z_{j}=1}$$ (4)

where the relationship of proximity in estimated propensity scores, "≅", is operationalised by a matching algorithm. Note that is not sufficient to multiply *ps* by r for the households that purchased fuel, to recover the distribution of consumption rates. For households that purchased are systematically different from motorist households that did not, having on average higher purchase probability and therefore higher probable consumption. PSM corrects for this difference and enables a full dataset of consumption rates to be estimated.

The matched, purchasing households thus provide an estimate of the set of unobserved values of *r*. The quality of these estimates, given (2), will depend on both sample size and the quality of the estimated propensity scores. Here *r* represents the quantity purchased conditional on a purchase occurring in the diary window. We refer to *r* as the ‘quantity at the pump’ to distinguish it from the consumption rate, *c*. How long a household takes to consume a given quantity is inversely proportional to its probability of purchasing. Each quantity is therefore multiplied by the corresponding estimated *ps* to yield an estimated rate of consumption, $\hat{c}$. That is, values given by

$${\hat{c}}_{i}=\begin{array}{r} {{\hat{ps}}_{i}(X).r}_{i}\;\mathrm{if}\;Z_{i}=1\\ {{\hat{ps}}_{i}(X).\hat{r}}_{i}\;\mathrm{if}\;Z_{i}=0 \end{array}$$ (5)

constitute the estimated distribution of consumption rates. Although $\hat{c}$ and $\hat{r}$ are subscripted it is important to realise that a given imputed value is not an estimate *for that household*, since each value of the scalar *ps*(**X**) is associated with a distribution of realisations of the vector **X**, not a specific configuration. At each propensity score, that is, there is still heterogeneity, but it is unrelated to Z. PSM therefore results in group-level matching: a set of households is identified with a covariate structure which is expected to be identical to that of the Z = 0 households.

Our application of PSM to infer the distributions of $\hat{r}$ and $\hat{c}$ is distinct from use of PSM for causal inference in observational studies. Firstly, in the latter context inferences from PSM generally only concern a mean, usually the mean effect of some intervention, rather than individual effects. This is because each effect is the difference between two potential outcomes, and one of these is unknown for each unit. Individual-level matching would be required to estimate individual effects and quantiles of the distribution. In the present setting, only one potential outcome is of interest, and it is unknown only for a subset of units. Secondly, in the causal inference problem the propensity score is only used to match units, whereas here it is used both for matching and to discount values for purchase infrequency. This implies a stronger condition for $\hat{ps}(X)$ to satisfy, since (2) can be satisfied even if there is omitted variable bias in the estimation of the probability of purchase. Suppose for example that all households purchased the same quantity (r) whenever they bought a particular good. Then (2) would be always be satisfied regardless of the quality of the propensity score model. We therefore make explicit a distinct assumption:

$$\hat{ps}(X)\;is\;an\;unbiased\;estimator\;of\;p(Z=1)$$ (6)

(6) implies (2) and (3), since if (6) holds there is (for example) no omitted variable bias, so there are no unobserved determinants of Z correlated with **X**, and therefore no such determinants of *r* and Z.

## Estimating the distribution of fuel consumption using PSM and the UK National Travel Survey

### Extent of infrequency of purchase

We consider data from the UK National Travel Survey (NTS), pooling data for years 2002–2008 to achieve a large sample size. NTS data in the unbanded format used here can be obtained on request and under license through the UK government’s Department for Transport, at NATIONAL.TRAVELSURVEY@dft.gsi.gov.uk. The NTS is ideally suited for study of infrequent purchase for the following reasons. Firstly, given its diary window of one week many households do not purchase fuel. Secondly, it also records annual mileage for each vehicle in the survey interview, which provides a crude proxy for fuel consumption. Finally, the data concerned are policy-relevant, particularly for environmental and energy policy, so practical consequences of the data problem are salient.

**Fig 1:**
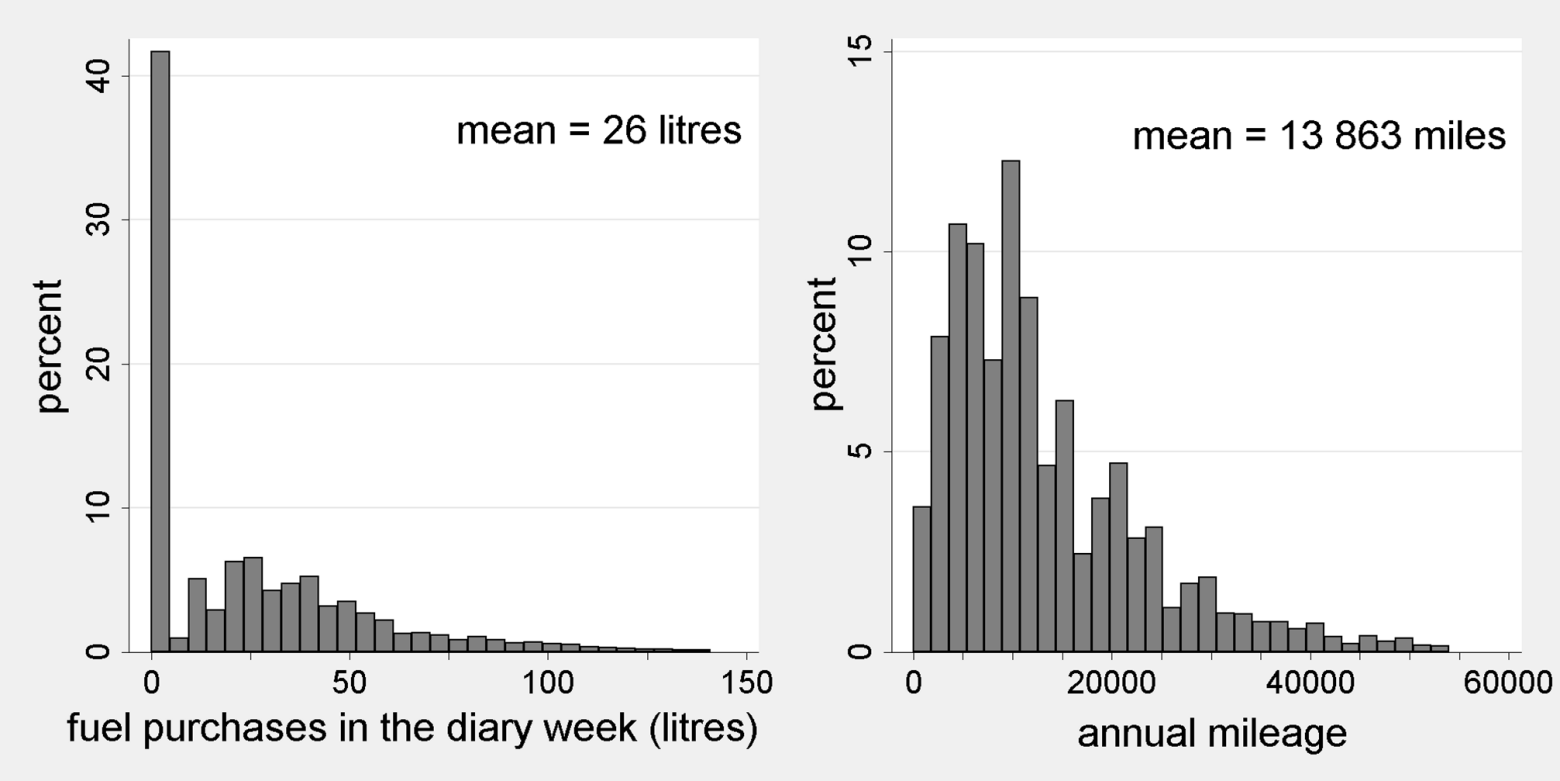
Sample distributions of motorist households’ fuel purchases and mileage, NTS 2002–2008. Notes: 1. The NTS reports mileage separately for each vehicle. The figure is obtained by aggregating over vehicles. 2. Censored at the 99^th^ percentile.

Concerning the extent of infrequent purchase, the sample comprises a total of 57,069 fully-cooperating households. Of these, 42,712 have vehicles, either cars, vans or motorbikes, but 17,485 (41%) did not buy fuel during the diary week. Only 70 vehicle-owning households actually report zero annual mileage. So only around 0.2% of motoring households in the sample should have no fuel consumption and almost all the recorded zeroes result from infrequency of purchase. Histograms of the diary data and mileage data are shown in Fig 1 below. The diary data show a spike at zero and an extended tail to the right of the mean.

We have no reason to believe the mean purchase is a biased estimator of the mean consumption rate (n2). Given the mileage data, however, the distribution of fuel consumption rates cannot resemble that in the left histogram of Fig 1. We anticipate a strong, direct relationship between the true distribution of mileage and the true distribution of fuel consumption rates. For, given the fuel efficiency of a vehicle, there is a determinate quantity of fuel required for a given journey. Consumption rates should therefore exhibit a distribution resembling that in the right histogram. The mileage data are not unproblematic, however, as the distribution has modes at multiples of 5000 miles, arising from over-reporting of salient numbers.

One could estimate fuel consumption directly from the mileage data, but there are serious disadvantages to doing so. Firstly, the NTS contains only discrete information relevant to the fuel efficiency. The relevant variables are a binary indicator of engine size (>1500 cc), a fivefold categorisation of vehicle type, and fuel type (diesel versus petrol) for each vehicle. Whereas for any given vehicle annual mileage we can expect a continuous distribution of fuel consumption rates. Secondly, the salient number bias would produce a multi-modal distribution of $\hat{c}$. Our strategy instead is to use the mileage variable as one resource for matching-based estimation amongst other covariates.

Table 1 below sets out the occurrence of purchases in the sample by equivalised income, using the ‘square root scale’ [20]. Whilst vehicle ownership is less common amongst less affluent households, the likelihood of non-purchase given ownership is higher. This implies that the divergence between the sample distribution of fuel purchases and that of the latent variable *c* is greater amongst less affluent motoring households. However, the problem is pronounced everywhere. Amongst the top income quintile, for example, 1/3 of motoring households have no recorded purchase and purchases exceed weekly consumption rates by a mean factor of ~1.5. This is calculated as the reciprocal of the mean purchase probability (= 1/(1–0.335) since 33.5% of motorist households in the top income quintile purchase no fuel, from Table 1).

**Table 1:** Extent of infrequency of purchase by income quintile.

Quintile of equivalised income	% of households with motor vehicles	% of motoring households with no fuel purchase
1	35.9	56.3
2	69.4	49.1
3	83.7	42.1
4	93.2	35.4
5	94.3	33.5

Notes

1. motoring households are defined as those owning at least one motor vehicle (car, van or motorcycle).

### Using PSM to recover ‘quantities at the pump’

The PSM is conducted using the vehicle-owning households only. Matching with replacement was applied, with a caliper of 0.01, using the psmatch2 routine in STATA [21]. In this approach, $\hat{ps}$ is the fitted value of a probit regression model. Each household which did not buy fuel is matched using $\hat{ps}$ to one that did, but the same match can be used more than once. This procedure is heterogeneity-preserving, which is appropriate here since we are attempting to recover an entire distribution.

**Fig 2:**
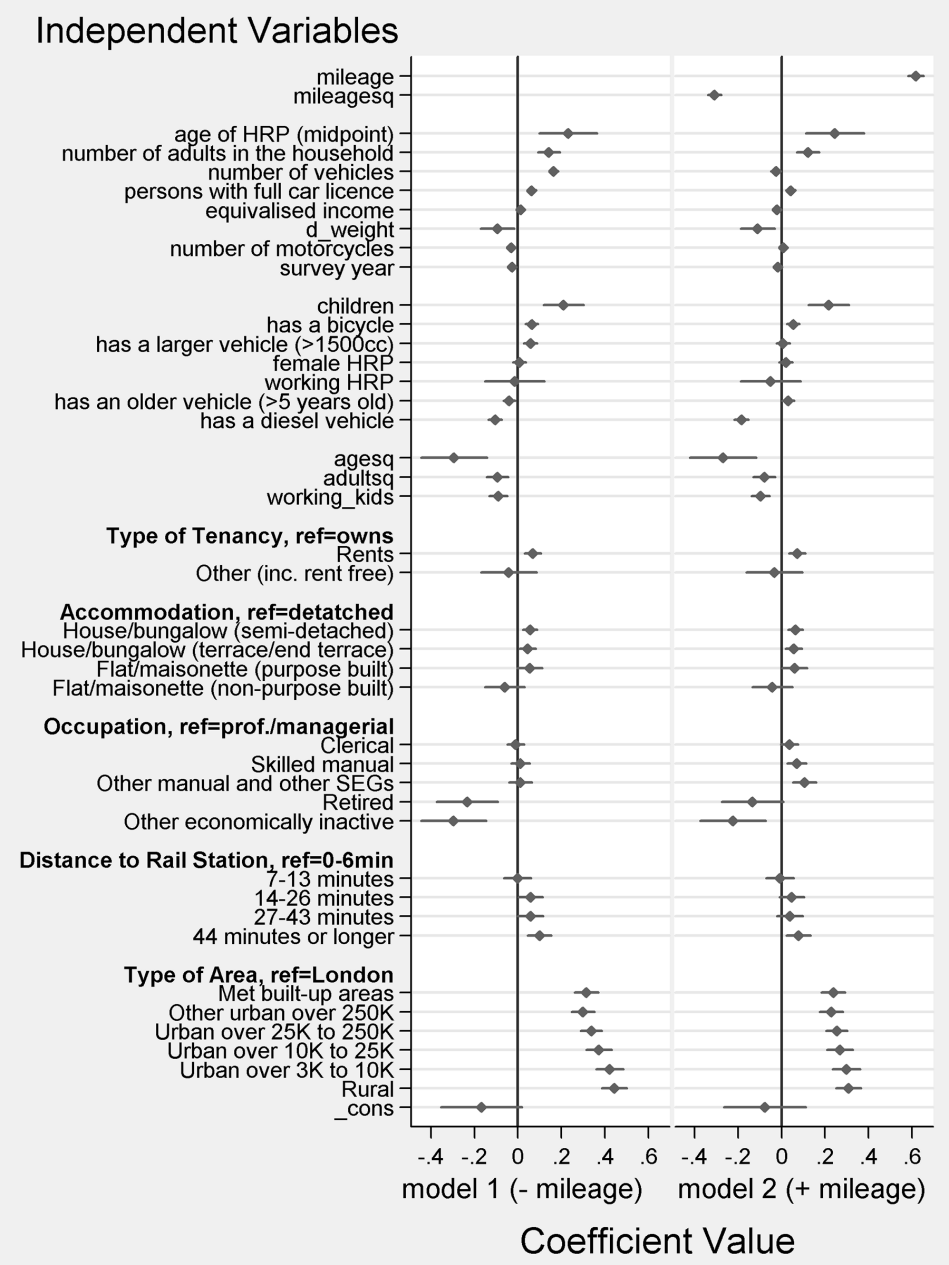
Coefficient plot of probit regression models of fuel purchase in the diary week. Notes. 1. Probit coefficients are shown as diamonds with lines representing confidence intervals. 2. Continuous regressors are standardised to have mean zero and unit variance. 3. Household-level variables are derived from individual- and vehicle-level data (authors’ calculations). 4. A dummy for each top-coded variable is included in the estimations but not shown. 5. For model 1 McFadden’s pseudo r-sq (adjusted) = 0.053, count r-sq (adjusted) = 0.11 and Log-L = -27250.28. Model 2 has 2 additional parameters; McFadden’s pseudo r-sq (adjusted) = 0.078, count r-sq (adjusted) = 0.16. and Log-L -26534.82. The LLR statistic is therefore 1430.92~χ^2^(2); p<0.001.

Two probit models were developed. The results are shown in a coefficient plot [22] in Fig 2 below. Model 1 makes full use of relevant covariate information in the NTS excluding the annual mileage variable. Although the NTS has not been collected to estimate fuel purchase propensity, it provides a rich set of relevant variables. We exclude mileage to see how the PSM-based imputation fares in the absence of a proxy for the imputed variable, since this will be the usual research situation. Square terms for age and numbers of adults are included, plus an interaction term for working households with children, since these were found to improve goodness of fit and matching quality. Model 2 simply adds the mileage variable as a regressor. This should help to correct for missing covariate information.

**Fig 3:**
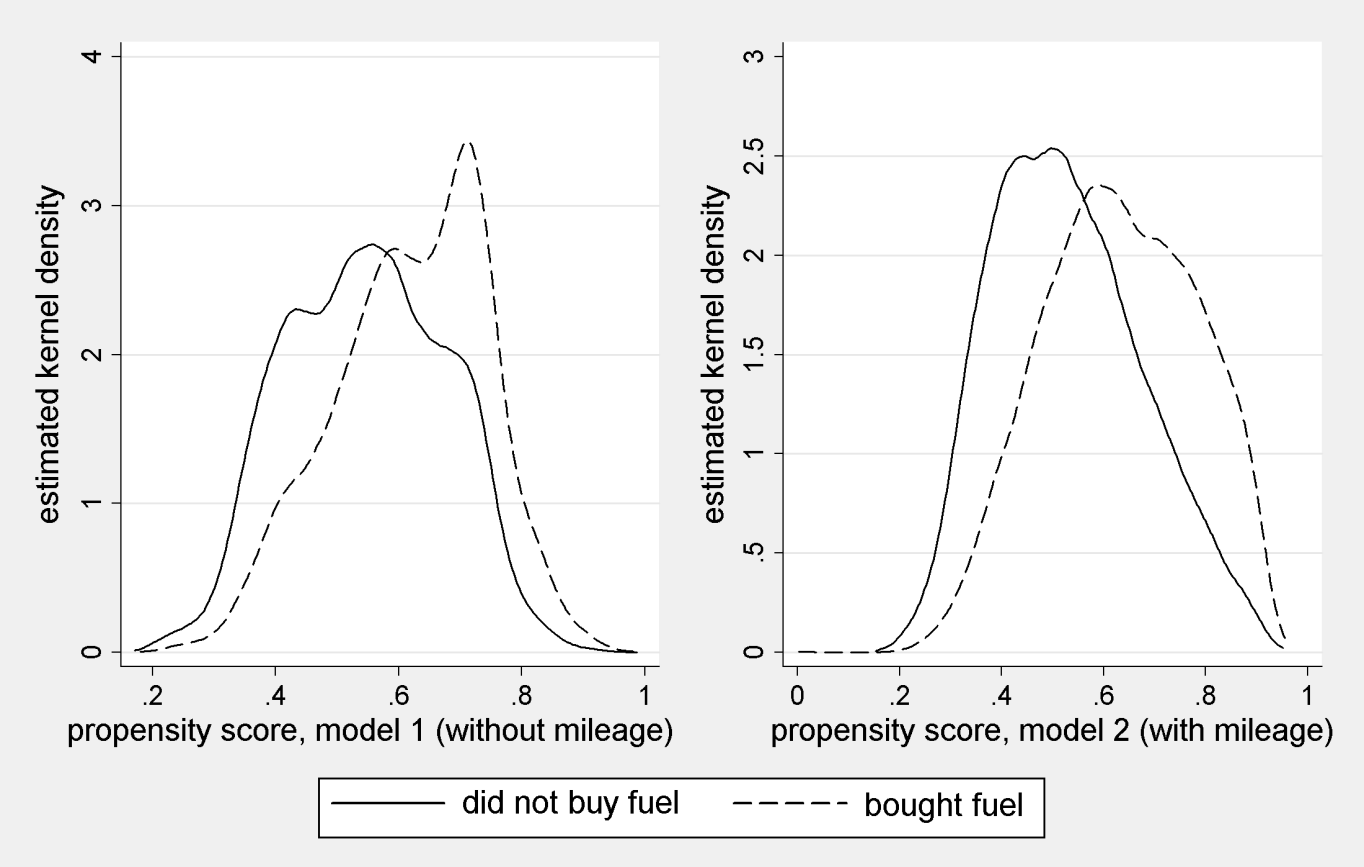
Estimated propensity scores: kernel density estimates. Note: Epanechnikov kernel.

Fig 3 below shows the distributions of $\hat{ps}$ in models 1 and 2, respectively, using kernel density plots. Common support is approximately satisfied. For there are no unmatched households under model 1, and under model 2, just 3 households are unmatched because of the 1% $\hat{ps}$ caliper we apply, and dropped. We regard this proportion as negligible.

The pronounced multi-modal distribution of model 1 propensity scores (Fig 3) is attributable to particular constellations of covariates with high-valued regression coefficients: two adult, two car, rural households with children, for example. Within such groups the distributions are approximately unimodal.

**Fig 4:**
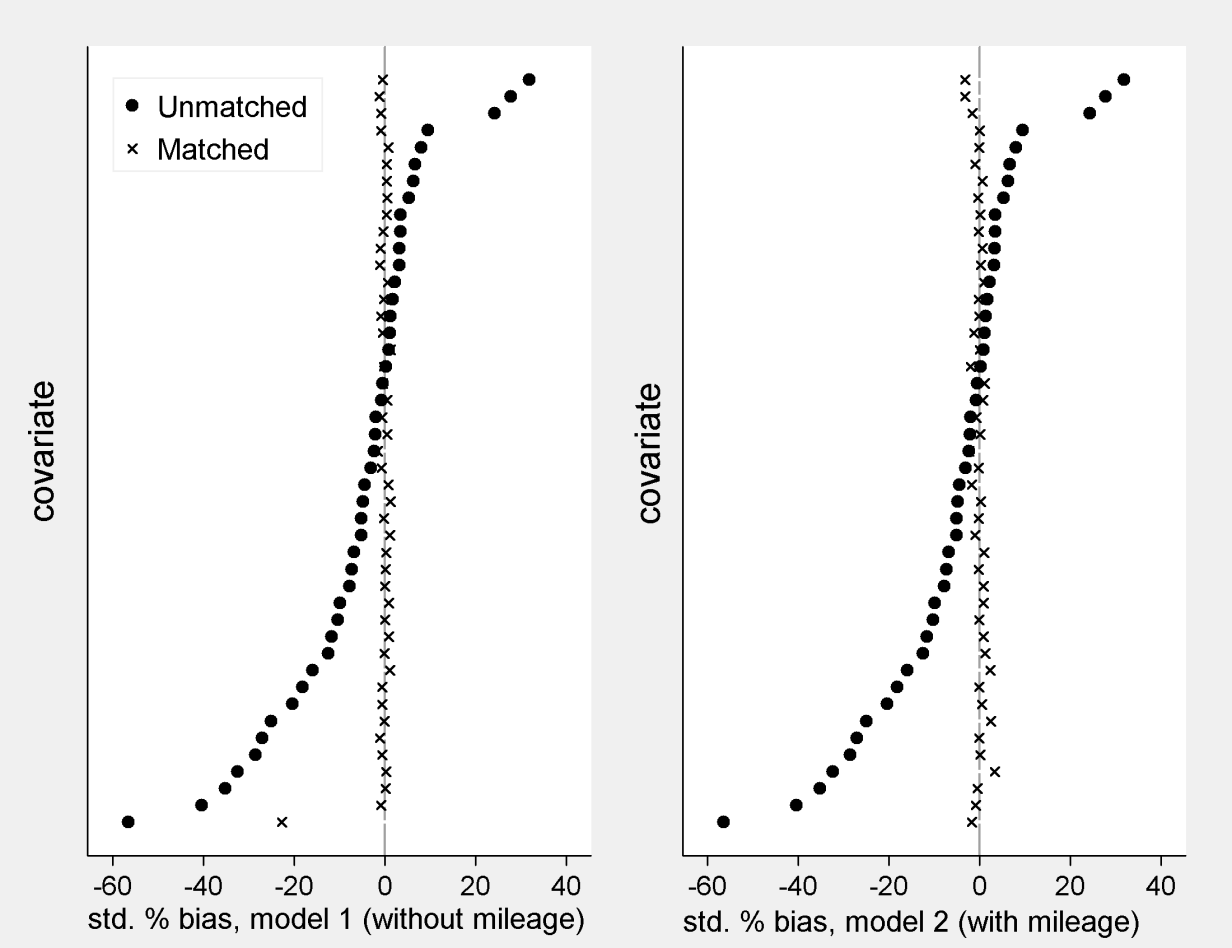
Standardised percentage bias between covariates in Z = 0 and Z = 1 households. Notes. 1. Covariates are those shown in Fig 2, less square and interaction terms, plus topcode dummies. 2. The mean, median and maximum absolute standardised % bias are 1.1, 0.6 and 22.7 for model 1 and 1.0, 0.7 and 3.3 for model 2.

Model 1 shows results which are generally in line with expectations, with positive coefficients for the number of driving licence holders, adults and children, the number of vehicles, distance from a train station and rural location, for example. Negative coefficients for diesel and motorcycles presumably reflect fuel efficiency. Model 1 performs poorly though, in terms of balance between the matched groups on the annual mileage variable. A visualisation of covariate balance is provided in Fig 4 below. Standardised percentage bias [23] is shown before and after matching for each coefficient, in order of pre-matching bias.

Covariates that are included in the regression have very low standardised biases (less than 2%). (Austin [24], for example, reports that standardised bias of less than 10% are regarded as low in applied work.) However, mileage, the coefficient at the bottom, shows the highest bias, exceeding 20%. Using $\hat{ps}$ from model 1, therefore, we obtain matched groups with significantly different mean mileage and, therefore, systematically different actual *ps*. This violates our requirement (6).

In model 2, the coefficient on mileage dominates the regression. Given the physical relationship between mileage and fuel consumption this is unsurprising. Since many of the independent variables are determinants of mileage, some coefficients change sign or become insignificant. Comparing (nested) models 1 and 2, conventional model selection criteria favour model 2 (note 5 to Fig 2).

Both models return seemingly “low” values of pseudo r-squared, but given the inherently stochastic data this does not evidence poor predictive performance. To illustrate this, recall that 60% of vehicle owners purchased fuel in the randomly-determined diary week, so that the sample mean purchase frequency is approximately 6 out of 10 weeks, a probability of purchase of 0.6. Two individuals with this purchase frequency will have identical in-sample behaviour only with probability 0.52 (= 0.6^2^ for purchase + 0.4^2^ for non-purchase). Therefore even a model which predicts purchase probability perfectly would be unable to predict whether a household actually purchased fuel across much of the dataset. Our objective for the PS model is to predict the purchase probability, not the act of purchase. For comparison, another context where event predictability is limited is nonresponse models, where values of McFadden’s pseudo r-squared well below 0.1 are quite normal [25, 26].

Whilst pseudo r-squared retains a comparative value for model selection, the covariate balance achieved is important in an absolute sense. Fig 4 indicates that the two groups are now well-matched on mileage, with only a slight worsening of the bias metric on the other independent variables. From this point on we therefore concentrate exclusively on model 2 estimates in the remaining figures and substantive discussion, and make reference to model 1 only for methodological purposes. A counterpart to each figure below is presented in the Supporting Information using model 1 estimates for comparison (Figs A-E in S1 File).

**Fig 5:**
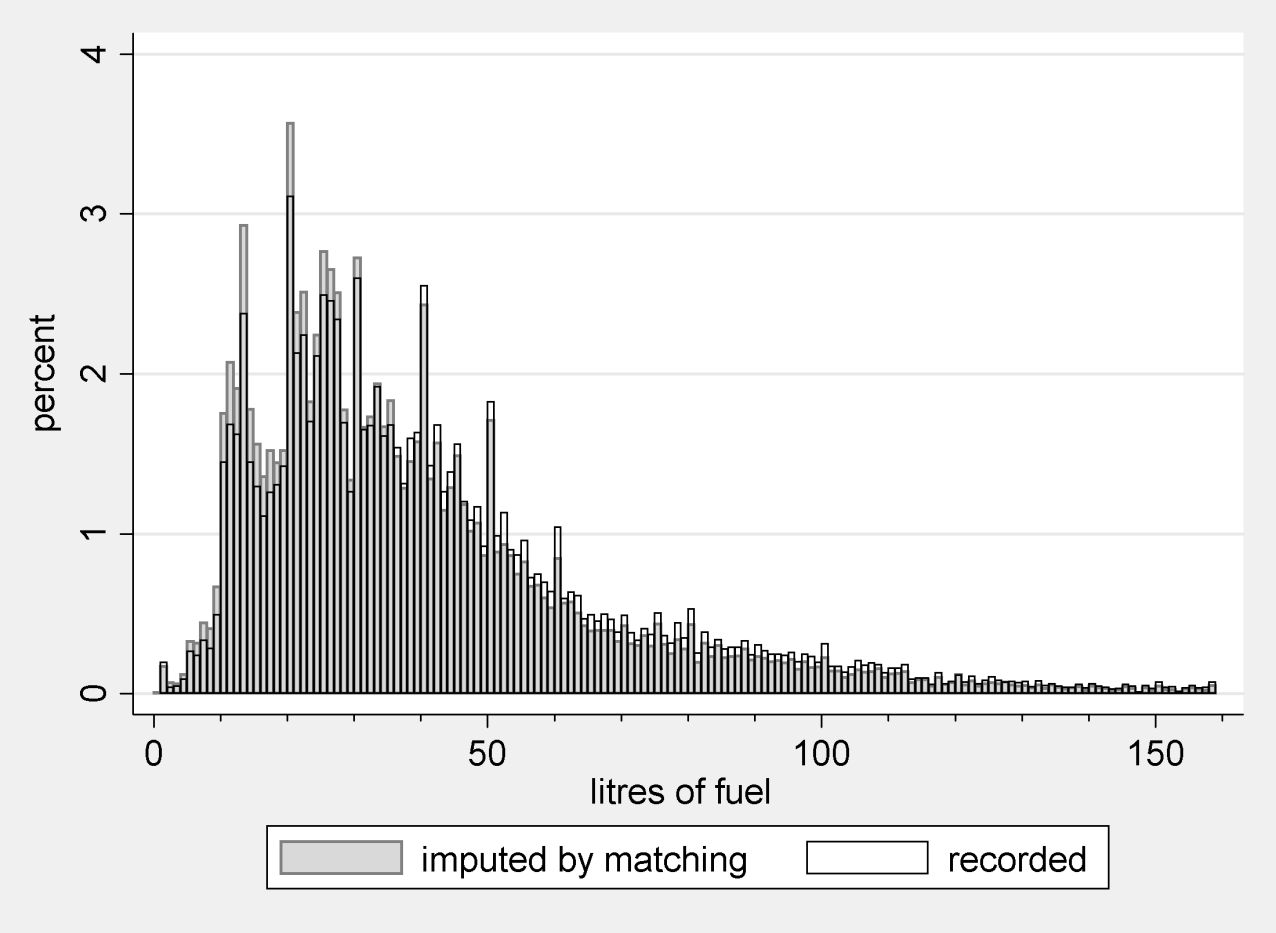
Quantities at the pump (litres) derived from PSM using model 2.

Having constructed the matched groups using PSM (with model 2), we take values for $\hat{r}$ from the matched set of Z = 1 households as stipulated in Eq (4). Thus, for households observed to buy fuel, we have recorded values of *r* and for Z = 0 households we have PSM estimates of quantities they would have bought, had they made a purchase, $\hat{r}$. Values of *r* and $\hat{r}$ are shown in Fig 5 below.

Fig 5 shows greater frequency of lower quantities amongst non-purchasing households. This is consistent with the difference in propensity scores between the two groups (Fig 4), and an association between Z and *r* prior to controlling for **X**. Also noticeable is the pronounced multi-modality of the distribution, with modes at multiples of 10 litres. Presumably this reflects a combination of over-reporting, and actually purchasing, salient numbers. Modes at 12–13 and 24–26 litres may be explained as follows. From 2002–2005, the price of petrol was roughly £0.80p per litre [27]. Thus, each £10 spent on petrol would result in a purchase of around 12.5 litres for half the period under consideration.

Fig 5 also illustrates the heterogeneity-preserving quality of the matching-based imputation procedure. The same pattern of modes at salient numbers is evident for both observed and imputed purchases.

### Estimated fuel consumption rates

**Fig 6:**
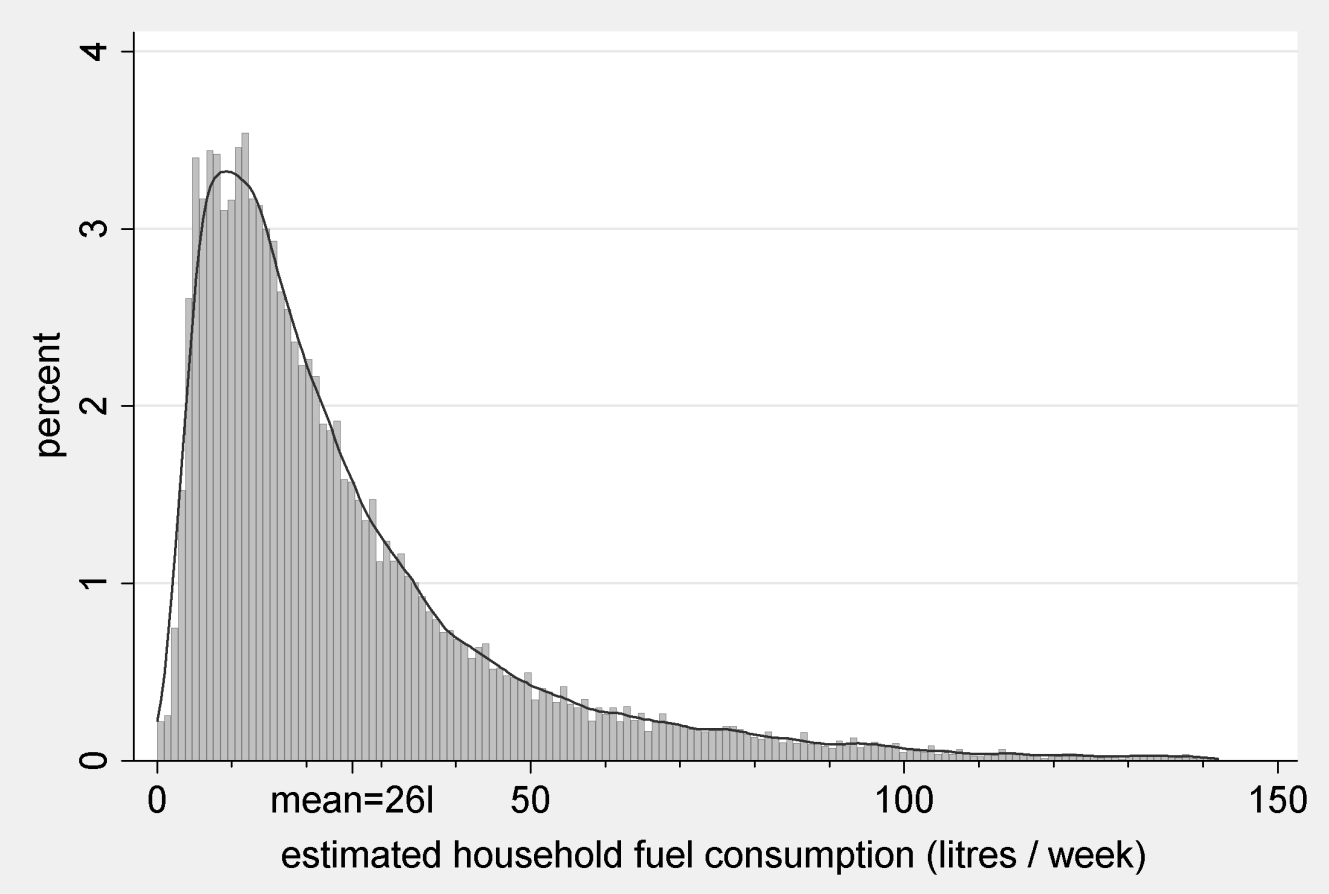
Preferred estimates of fuel consumption rates, derived from model 2. Notes 1. Kernel density estimates (Epanechnikov kernel) overlaid. 2. Excludes top percentile.

Having derived quantities at the pump, the next step is to multiply each quantity by its associated propensity score to obtain estimated consumption rates, $\hat{c}$, as specified in Eq (5). The resulting estimates are shown in Fig 6 and summarised in Table 2 below, alongside estimates using $\hat{ps}$ from model 1, the diary fuel purchase and annual mileage variables.

**Table 2:** Estimates from PSM based imputation, recorded fuel purchase and mileage.

Statistic	Fuel purchase in diary week (litres)	Annual mileage (thousand miles)	Weekly fuel consumption from PS model 1	Weekly fuel consumption from PS model 2 (preferred)
percentile 1	0	0.7	3.1 (0.11)	2.6 (0.10)
5	0	2	5.1 (0.08)	4.6 (0.08)
10	0	3	6.7 (0.09)	6.1 (0.09)
25	0	6	11.2 (0.12)	10.4 (0.12)
50	18	10	19.1 (0.15)	18.3 (0.15)
75	40	18	32.7 (0.26)	32.5 (0.25)
90	67	28	54.5 (0.55)	55.3 (0.51)
95	90	35	71.2 (0.75)	75.6 (0.74)
99	142	54	115.1 (1.77)	124.5 (2.13)
mean	26.0	13.7	26.1(0.18)	26.1 (0.16)
std	33.1	11.3	23.4	25.2
skewness	2.4	2.1	2.9	3.0
N	42600	42707	42598	42595

Notes

1. Bootstrap standard errors in parentheses, with 1000 repetitions. See main text and note 8 for discussion.

2. N varies across columns because of missing NTS data. In addition, 3 observations cannot be matched using model 2 under the 1% *ps* caliper restriction applied.

Standard errors for fuel consumption in Table 2 are calculated by bootstrapping, incorporating variation associated with the *ps* estimation and matching. Regular bootstrapping in this context fails to reproduce the distribution of times a unit is used as a match, *f*_*i*_ [28]. We avoid this problem by adding a small random error, *e*, to $\hat{ps}$ after drawing each bootstrap sample but before conducting the *ps* matching. This resolves the problem in theory, given values of *e* small enough for Eq (4) still to hold but large enough to perturb the match selected.

**Fig 7:**
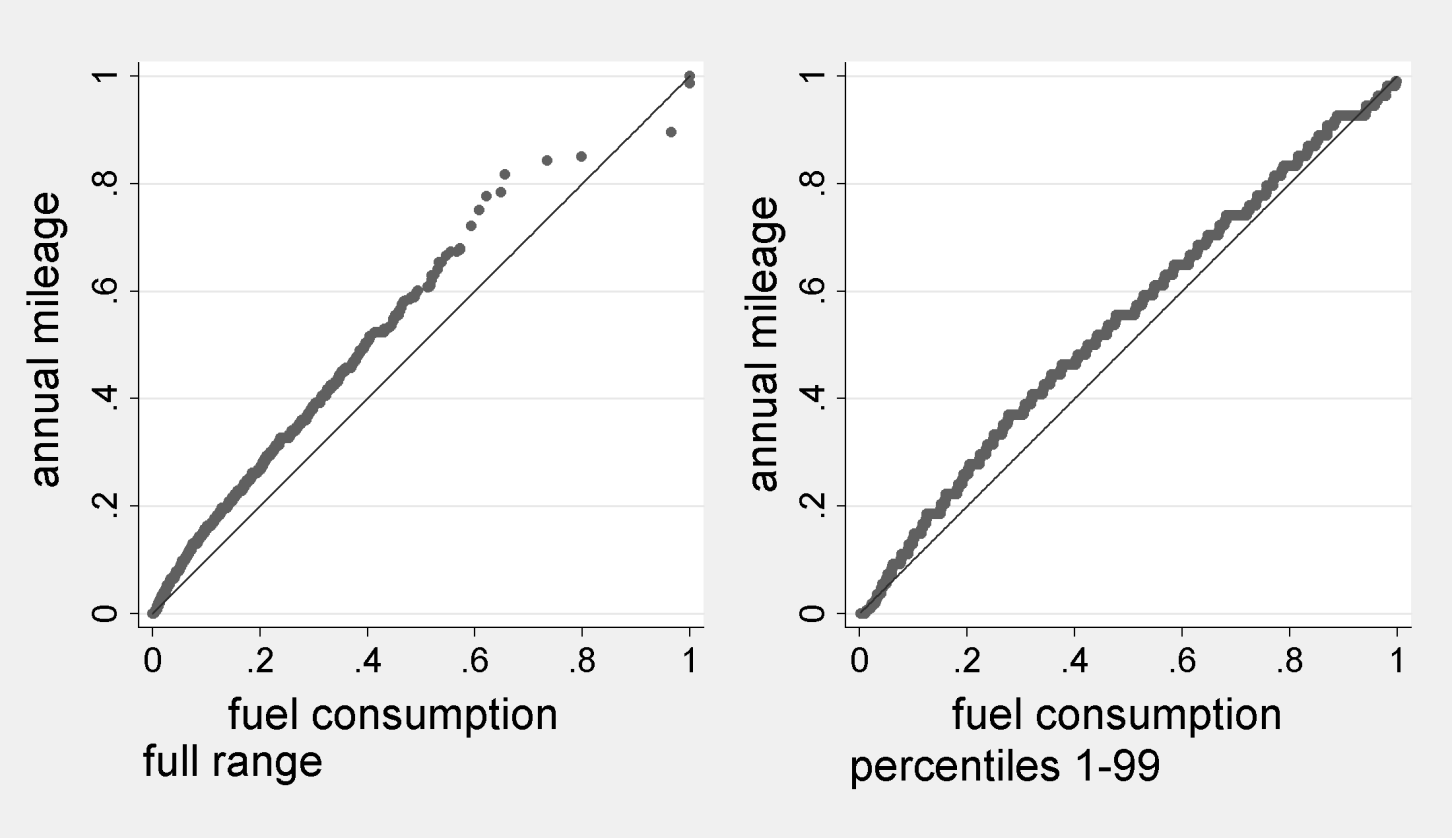
Quantile-quantile plots of estimated fuel consumption rates against recorded annual mileage. Note Values have been divided by the maximum of the range.

The distribution used was *e~*N(0, 1/30625). We selected parameters for *e* which approximately reproduce the distribution of *f*_*i*_ without detriment to the standardised bias metric of matching quality, by trial and error (Table A in S1 File). We also tested our bootstrap procedure using Monte Carlo simulation (Table B in S1 File). The bootstrap standard errors for the mean and quantiles of the distribution approximate standard deviations of the corresponding variables derived using simulated samples, but those for standard deviation and skewness do not, a problem which seems attributable at least in part to skewness of c (notes to Table B in S1 File). We therefore include standard errors only for the mean and percentiles in Table 2. We offer the following observations on the quality of the preferred estimates (Table 2, column 4). $\bar{\hat{c}}$ approximately equals the mean fuel purchase (26.06 litres versus 26.03 litres respectively), as required. At the same level of granularity, the multimodality of Fig 5 is absent from Fig 6, which is reassuring since it is unlikely that *c* is affected by salient number biases. The distribution also appears plausible judging the mileage proxy. Let Q1, Q2 and Q3 denote the 25th, 50th and 75th percentiles of a distribution respectively. The proportional relationships Q1/Q2 and Q1/Q3 are identical for mileage and $\hat{c}$ to one decimal place. For a more detailed comparison we present quantile-quantile plots in Fig 7, normalising by dividing each value by the maximum of the variable.

The left plot of Fig 7 shows that the quantiles of $\hat{c}$ are located somewhat lower in their range, than are quantiles of mileage. For example, Q3 fuel consumption is .07 of the maximum value (32.5/469). Q3 mileage is .12 of the maximum value (18,000/153,000). Thus one point in the above plot is (.07, .12). Since for both variables the 99^th^ percentile is less than 0.35 of the maximum value, the right plot is drawn for percentiles 1–99 only. This confirms that values are somewhat more concentrated at lower areas of the range for fuel consumption, consistent with the difference in skewness shown in Table 2. This may be associated with features of the distribution of vehicle fuel efficiency. However, in both cases the plots do not deviate dramatically from the 45-degree line and the larger deviation concerns the top 1% of observations.

### Estimated fuel consumption rates without a proxy variable

Our preferred estimates derive from model 2, which includes a proxy (mileage) for the target variable (fuel consumption). Normally a researcher would lack this, so it is appropriate to reflect further on the quality of the estimates obtained without the proxy, using model 1. The corresponding quantile-quantile plots using estimates from model 1 are very similar to Fig 7. They show a greater deviation from the 45 degree line for the full range plot, and less deviation from it for the first 99 percentiles (Fig A in S1 File). From Table 2, although the percentiles obtained under the two models are generally significantly different, this is attributable to the large sample size. The absolute differences in $\hat{c}$ are fractions of a litre per week excepting at the upper tail, and the mean is approximately the same. Thus, even without the mileage proxy included in the *ps* estimation the estimates seem plausible.

PSM using model 1 matches households with systematically different actual fuel use, since they have systematically different mileage (Fig 4, left). Ignorability, (3), may approximately hold without including mileage in **X**, however, since it is a condition on *r*, not *c*. Consistently with this, the distributions shown in Fig 5 are very similar if we use model 1 estimates (Fig B in S1 File; the estimates differ by 1l at the median and 1.2l at the mean, about 3% in each case).

It therefore seems probable that under model 1, draws from very similar distributions of quantities occur to those under model 2. But the spread of $\hat{ps}$ under model 1 may be relatively narrow. This is consistent with the higher standard deviation of $\hat{c}$ under model 2 (Table 2), and the larger variance in $\hat{ps}$ (Fig 3). It seems from the plausibility of the model 1 distribution that the resulting errors are counterbalanced to a significant degree. This may not be surprising, given that with an appropriately designed and implemented sample survey we have an unbiased estimate of $\bar{ps}$ (= $\bar{Z}$) prior to any modelling of the purchase decision. The case studied therefore seems encouraging from the perspective of the researcher who lacks a proxy for the target variable. For it seems that omitted variable bias in the *ps* model may sometimes have little effect on the bulk of the estimated distribution. However, the two sets of estimates are more divergent at the highest quantiles of estimated consumption. This may be because model 1 underestimates the occurrence of households for whom *ps*>0.75 (Fig 3).

## Application to emissions reduction policy

### Estimation of UK household CO_2_ emissions from motor vehicles

Given increasing greenhouse gas concentrations in the atmosphere, it is interesting to consider the relevance of our results to discussions of household CO_2_ emissions, particularly since infrequent purchase has constrained their analysis. On UK households’ greenhouse missions, see for example Gough et al. [29] and Büchs and Schnepf [30]. We calculated CO_2_ emissions of each vehicle using the fuel purchase diary and DECC / DEFRA emissions factors [31], using separate figures for petrol and diesel. These figures were then aggregated to yield motoring emissions for each household. The resulting estimates suffer from essentially the same infrequency of purchase problem outlined above, and are treated in the same way. That is, we substitute the emissions quantity for each Z = 0 household with the value obtained for its *ps*-matched observation, using model 2 estimates, and then multiply each emissions quantity by its estimated *ps*. The resulting estimates, $\hat{d}$, are strongly isomorphic to $\hat{c}$, since emissions are simply a multiple of the amount of each fuel purchased representing its carbon content. Mean (median) annual motoring emissions over the period are calculated to be 2.4 (1.5)t CO_2_ per household, or 3.2 (2.2)t CO_2_ per motorist household.

Of particular interest is the estimated concentration of emissions, a notable study having reported that they are disproportionately accounted for by a relatively small group of high-emissions households [32]. We summarise estimated shares of vehicle CO_2_ emissions by (emissions) decile in Table 3 below.

**Table 3:** Decile shares of UK motorist households’ CO_2_ emissions from motor fuels.

Emissions Decile	% share (SE)	Cumulative % share
1	1.7 (.03)	1.7
2	2.9 (.04)	4.5
3	4.1 (.05)	8.4
4	5.2 (.05)	13.4
5	6.4 (.06)	19.6
6	7.9 (.07)	27.3
7	9.8 (.08)	37.0
8	12.5 (.11)	49.5
9	17.3 (.16)	66.9
10	32.4 (.34)	100

Note. Bootstrap standard errors in parentheses, with 1000 repetitions. See main text and note 8 for discussion.

This breakdown confirms the concentration of vehicle emissions, with an estimated 1/2 of motor fuel CO_2_ accounted for by the top quintile, and 1/3 by the top decile alone. The advantage of our estimates is that they are based on a national representative survey. Brand and Boardman used a local sample survey conducted in Oxfordshire coupled with an online survey, so the estimates have an ambiguous geographic and statistical status. The authors also report a ratio between the top and bottom quintiles of 15:1. Our estimate for the UK is lower, but still remarkable, at 10.9:1 with 95% c.i. (10.5 ≤ *x* ≤ 11.2):1 (±1.96 x bootstrap standard error).

As Brand and Boardman [32] suggest, the policy implication of the high concentration of emissions is that reducing those of a relatively small proportion of (generally richer) high emitting households would be highly effective in terms of tackling overall emissions. In absolute terms the policies usually discussed, namely carbon taxes and carbon rationing, would both affect higher emitting households more, but operate regardless of income *per se*. How such policies would affect different income groups has therefore attracted much attention [33]. The literature has been unable to estimate the spread of policy impacts *within* different income bands, however, since the available national surveys are all affected by infrequent purchase. So although mean effects have been estimated by income group, it is not known how representative these are. That they may be heavily influenced by relatively extreme values is suggested by the high skewness of $\hat{c}$ in Table 2. For further insight we use our estimates of $\hat{d}$ and covariate information in a simple simulation of emissions reduction policy.

### Motor fuel emissions reduction policies

#### Static microsimulation

Ideally one would conduct a sophisticated policy simulation incorporating behavioural responses and a model of the economy [34]. Examples include REMI [35] for emissions reduction policies for the USA, and Comhar [36] for transport fuel policies in the Republic of Ireland. But that would constitute a complex study in its own right, and introduce many additional sources of uncertainty. Instead we use the simplest approach, static microsimulation to illustrate directly the value of our method.

Static microsimulation is an attempt to show effects of an intervention without taking possible behavioural change into account. It is a widely-used policy analysis tool, especially in research that examines possible distributional effects of taxes and benefits. Examples include applications of the European static microsimulation tax and benefit model Euromod [37–39], and studies estimating effects of financial instruments for energy or emissions reduction [40,41].

In essence, we calculate ‘who stands to lose how much’ under a policy. Such analysis is frequent in the media and offers a starting point for policy evaluation. It offers insights into policy resistance / acceptance, and is an important step towards analysis of regressivity. It provides insight into probable early effects, since behaviour and the economy take time to change. It can be especially informative if behaviours are shaped by physical infrastructures and/or social norms which usually change slowly, and if alternative travel modes are poor substitutes. Arguably, these are characteristic features of motor vehicle use, accounting for low price elasticity of fuel consumption, with a typical developed country study estimating around 2.5% reduction in fuel demand following a 10% increase in price after 1 year [42]. In our concluding section, we nonetheless go beyond static microsimulation to offer some reflections on how behaviour might react to different policy variants.

For recent discussion of emissions reductions policies, including implementation issues, see Sorrell [43]. Two such policies are considered here. The first is a carbon tax or tradeable ration / cap. Taxes and caps would have very important differences in practice, since taxes have uncertain impact on emissions, whereas a cap has uncertain effects on prices. But the two policies are analytically equivalent within our framework. Thus, we assume either that a tax is levied at a certain rate without behavioural response, or that fuel use is capped at current levels and price responds by an assumed amount because of scarcity at the margin as consumers have to buy permits. We will assume £100/tCO_2_ as the tax rate (or price increase). The second policy is the same tax or ration implemented in revenue- neutral form. That is, the carbon revenue is allocated to the households on an equal per-capita basis, with each adult aged 16 or over allocated an equal share. Tax and ration / cap variants of this policy are known as ‘tax and dividend’ [44] or ‘cap and share’ [36] respectively. A household’s net payment, *v*, is defined as its payment for carbon content of its fuel, *t*, minus its income from per-adult revenue shares.

Since our estimates are derived using matched groups, we cannot identify a Z = 1 household a given Z = 0 household is matched to. We need household-level information though, to calculate outcomes by income decile and / or as proportions of income. We address this problem exploiting property (1) of *ps*: the structure of observed covariates in the matched controls, for a large sample and true *ps*, is identical in expectation to that in the original group. Income is an observed covariate, included as a regressor to calculate $\hat{ps}$. We therefore substitute the Z = 0 households for the matched controls, dropping the former from the dataset. In the next 2 subsections we report estimates of (net) payments under the two policies, expressed in absolute terms and as proportions of income. These are represented in the following variables:

$${\hat{t}}_{i}=100\;{\hat{d}}_{i}=tax(£)$$

$$\frac{{\hat{t}}_{i}}{y_{i}}=tax\;as\;a\;proportion\;of\;income$$

$${\hat{v}}_{i}={\hat{t}}_{i}-pm_{i}=rebated\;tax\;(net\;payment)$$

$$\frac{{\hat{v}}_{i}}{y_{i}}=rebated\;tax\;as\;a\;proportion\;of\;income,$$

where

$$p=\frac{\hat{100\sum_{i=1}^{n}{\hat{d}}_{i}}}{\sum_{i=1}^{n}m_{i}}(£)=the\;value\;of\;the\;per\;capita\;revenue\;share$$

$$m_{i}=the\;number\;of\;persons\;aged\;\geq 16\;in\;a\;household$$

and

$$y_{i}=equivalised\;household\;income$$

#### Effects of a carbon tax or ration of motor fuels on UK households

**Fig 8:**
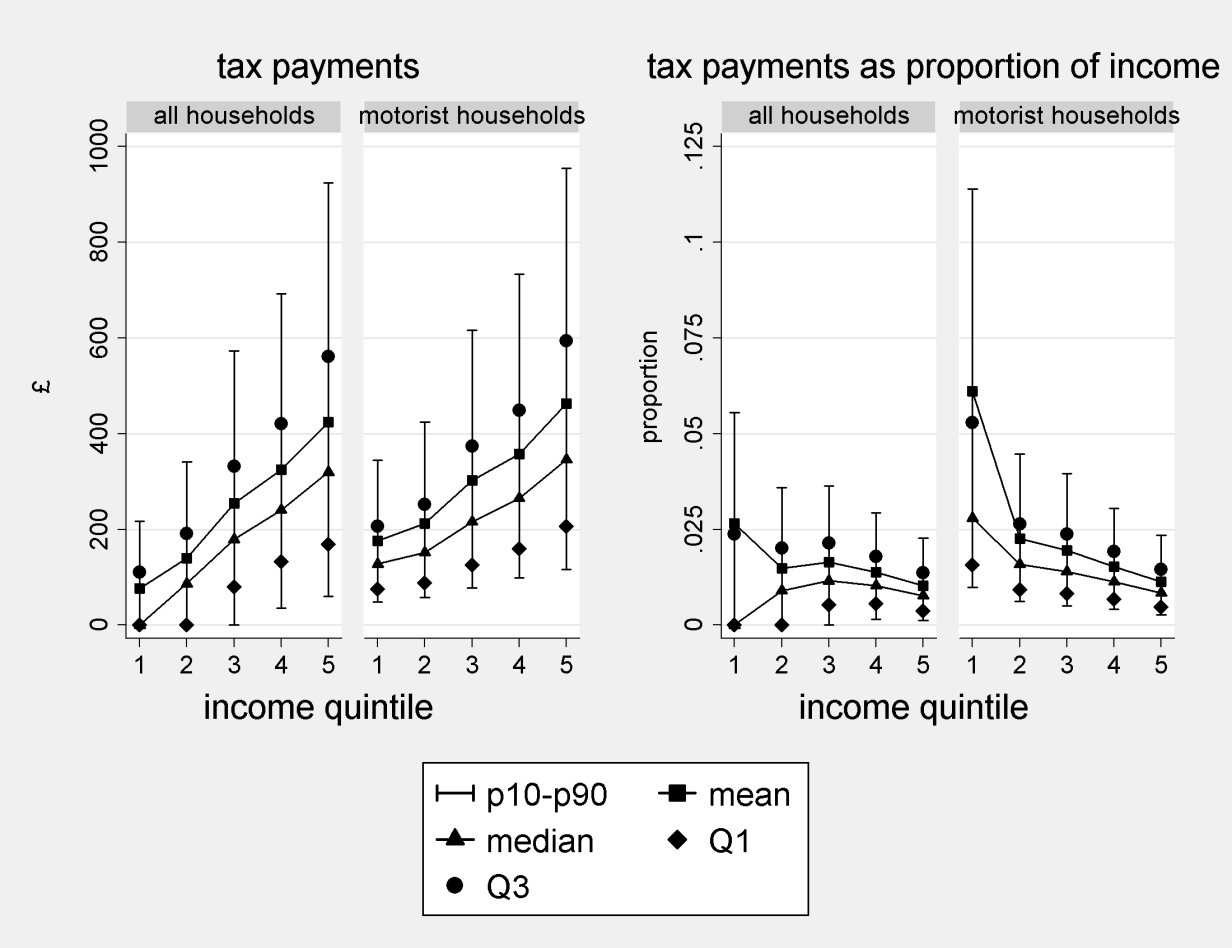
Estimated monetary effects of a carbon tax or ration at £100/tCO_2_. Note: ‘diary sample’ weights applied, recalculated for the pooled sample.

${\hat{t}}_{i}$ and $\frac{{\hat{t}}_{i}}{y_{i}}$ are shown using distribution plots in Fig 8 below. The plots show the 10^th^ and 90^th^ percentiles, Q1, Q2, Q3 and means of the estimate over quintiles of equivalised household income. The two measures of central tendency are shown connected to show the gradient across income quintiles. Although we stipulate a £100/tCO_2_ carbon price, since $\hat{t}$ is simply a multiple of $\hat{d}$, estimated effects at other prices can be directly inferred. For example, at £200/tCO_2_ each figure on the y-axes would be doubled.

A clear feature of $\hat{t}$ evident in the left-side plots is its consistent positive skew. For motorists, the mean payment exceeds Q2 by 34–40%, and in the first two quintiles is closer to Q3 than Q2. Thus, reporting on mean effects considerably amplifies the impact on a ‘typical’ household compared to the more representative and robust Q2 and seems particularly misleading at lower incomes. Another interesting feature of the distributions is the relatively low gradient between the 1^st^ and 2^nd^ income quintiles of motorists (2^nd^ plot from the left). This may be an indication that car use is mainly for essential journeys rather than leisure at lower incomes. The higher gradient between these quintiles in the leftmost plot reflects the increase in vehicle ownership with income.

The right hand plots show $\frac{{\hat{t}}_{i}}{y_{i}}$. The tax appears regressive amongst motorists (rightmost plot), with the gradient in incomes exceeding that in absolute payments. However, the mean proportional tax is closer to Q3 than Q2 for quintiles 1–3 indicating heavy influence by relatively extreme values. For the 1^st^ quintile, for example, it is approximately double the Q2 value of ~3%.

The picture is more complicated for the population as a whole (2^nd^ plot from the right), due to low rates of vehicle ownership in the 1^st^ and 2^nd^ quintiles. At the mean, the policy is estimated to be regressive, though $\frac{{\hat{t}}_{i}}{y_{i}}$ does not decline monotonically across quintiles. Our results at the mean contrast with an earlier claim in the policy literature, that taxes on motor fuels are progressive overall, and only regressive amongst motorists [45, 46]. This difference is likely to be attributable to increasing car ownership over time. According to the NTS, 44% of households in the lowest income quintile owned or rented a car over 2002–2008, up from 34% in 1995/1997 ([47] and own calculations). We estimate that evaluated at the median, the tax is progressive across quintiles 1–3 but slightly regressive across 3–5. Again, the mean proportional tax is closer to Q3 than Q2 for quintiles 1–3.

#### Effects of ‘cap and share’ or ‘tax and dividend’ for motor fuels on UK households

**Fig 9:**
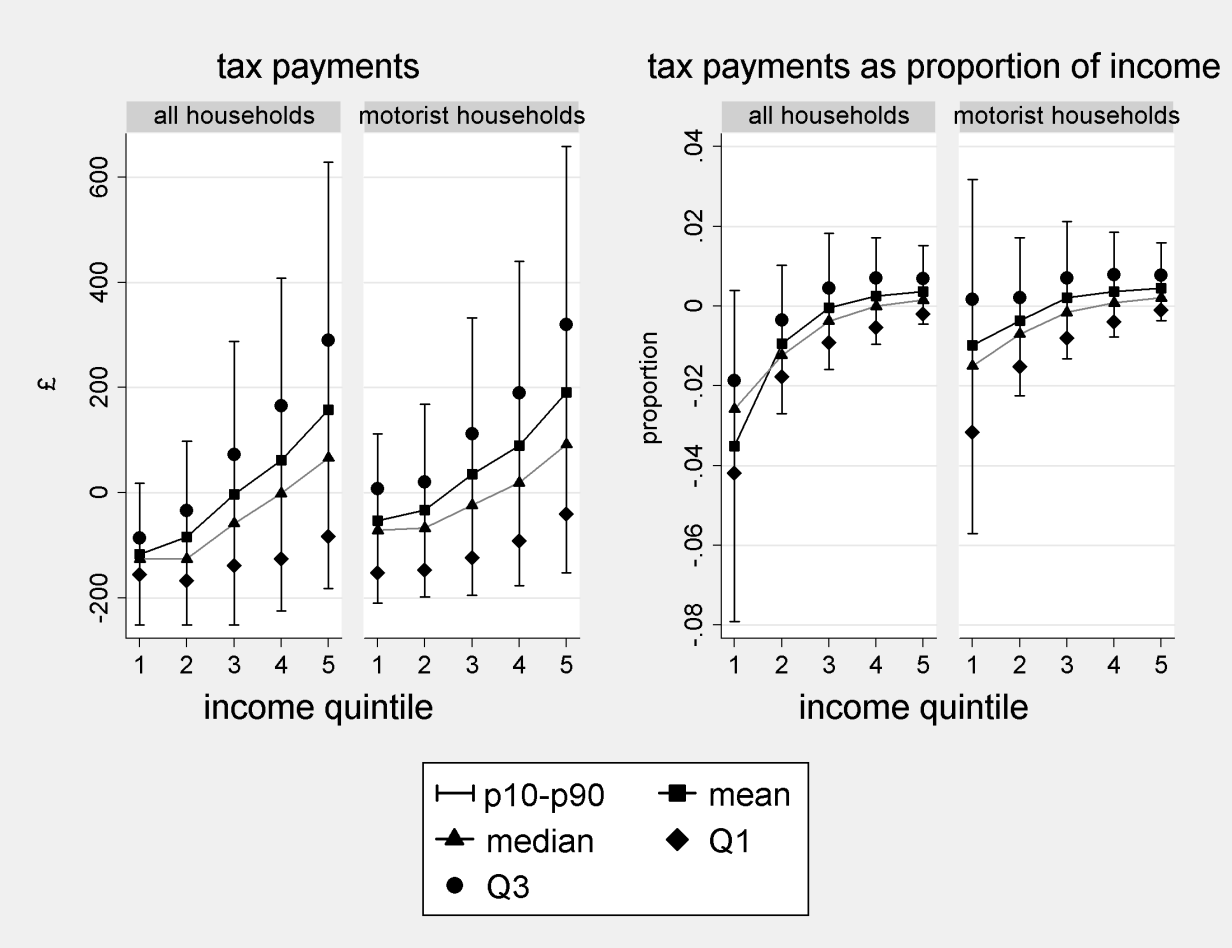
Estimated monetary effects of a ‘cap and share’ or ‘tax and dividend’ policy at £100/tCO_2_. Note: ‘diary sample’ weights applied, recalculated for the pooled sample.

${\hat{v}}_{i}$ and $\frac{{\hat{v}}_{i}}{y_{i}}$ are represented in Fig 9 below for a tax rate of £100/tCO_2_ rebated to the population. The value of the per capita payment at this tax rate is estimated at £127. Again, effects of different CO_2_ prices can be directly inferred by rescaling the axes. In these plots, in addition to the gradient, it is interesting to consider the predicted proportions of the quintiles or population that stand to win ($\hat{v}$<0) and lose ($\hat{v}$>0) financially.

Consider first the left-side plots, showing ${\hat{v}}_{i}$. Consistently with Fig 8, there is a low gradient between quintiles 1 and 2 in terms of absolute payments amongst motorists, reflecting broadly similar patterns of car use. The gradient between these quintiles in the leftmost plot (compared to the corresponding figure in Fig 8) is lower. This reflects fewer single-adult households in quintile 2 than in quintile 1 (39% versus 58%), and consequently more dividend payments, compensating for increased car ownership. There is again positive skew but less extreme that in Fig 8, with means slightly closer to Q2 than to Q3. Considering winners and losers, the mean household gains in quintiles 1–3 (all households) or 1–2 (motorist households), whereas the median household gains in quintiles 1–4 or 1–3 respectively. Overall, only the richest quintile are estimated more likely to lose than gain, and most motorist households are actually estimated to gain. Analysis at the mean conceals these features, which are highly politically salient.

Now consider the right-side plots, showing $\frac{{\hat{v}}_{i}}{y_{i}}$. In contrast to Fig 8, these show strongly progressive outcomes, with relatively large percentage gains at lower incomes, paid for by relatively small transfers from higher income households. In quintile 1 (all households), there is negative skew, which occurs because a relatively small group of households do extremely well proportionally under the policy: namely those without vehicles with very low cash incomes and large numbers of over-16s. The mean here is closer to Q1 than Q2. The other distributions exhibit positive skew. For example, the mean household in quintile 1 stands to gain an estimated 1% of income and the median household an estimated 1.5%, a proportional gain 50% higher.

Lacking estimates of quantiles because of infrequency of purchase may therefore have appreciable impact on policy analysis. Previous studies of emissions reduction policies for motor fuels had to rely on mean consumption rates, including means for different income bands. But a carbon tax or cap appears considerably more regressive, and its revenue-neutral counterpart less progressive, when evaluated at the mean rather than at the median. In short, the policy seems better with the quantile information. The picture is not uniform however, since not all the estimated distributions exhibited positive skew. We judge that the overall pattern could not be predicted simply by inspection of the underlying variables.

The p10-p90 ranges for the lowest income households in the rightmost plots of Figs 8 and 9 suggest extreme heterogeneity, which could be politically problematic since extreme cases often receive prominent media attention. These estimates are probably affected by further data limitations, however, since low income households may rely heavily on the benefits system, which is not accounted for in the NTS. Additional data collection would presumably be necessary to better evaluate outcomes at the lowest incomes.

Finally, we note that conducting the same policy simulation using propensity scores from model 1 to estimate $\hat{d}$ produces almost identical results across the bulk of the distribution. The graphs obtained corresponding to Figs 8 and 9 are visually distinguishable only at the 90^th^ percentile for quintiles 4 and 5 in the left side plots (Figs D and E in S1 File). This is consistent with our earlier observation that estimates from the two models of fuel consumption rates differ substantively only in the right tail of the distribution.

## Conclusions

A simple method was presented whereby propensity scores can be used to adjust a variable affected by a short observation window in sample surveys, a longstanding problem precluding distributional analysis. First, match each Z = 0 unit to a Z = 1 unit on the $\hat{ps}$ to obtain $\hat{r}$ for the former. Second, multiply each value of *r* (if Z = 1) and $\hat{r}$ (if Z = 0) by $\hat{ps}$ to obtain estimates of the latent variable *c* of interest. The problem and method were illustrated using the UK National Travel Survey, which contains a proxy (annual mileage) for the affected variable (fuel purchase). The resulting estimates of fuel consumption rates are plausible judging by the distribution of household annual mileage calculated from the same survey. Estimates obtained without recourse to the mileage proxy are also plausible, differing substantially from our preferred estimates only in the upper tail of the distribution. This is encouraging, since a proxy for the target variable will not normally be available.

The method was then applied to conduct a static microsimulation of two emissions reduction policies for motor fuels, supposing a carbon price of £100/tCO_2_. Such exercises have previously had to rely on estimates of mean effects. We judge that estimating entire distributions of effects shows the policies in a more favourable light. The distributions appear to be highly skewed, influencing the mean appreciably, but not always in a consistent direction. This information is timely given the outcome of the recent COP 21 meeting, which agreed targets and aims for curtailment of global warming, but did not agree any emissions reduction policies to achieve these.

A simple carbon tax or ration / cap would be regressive amongst motorists, but appears less regressive evaluated at the median than at the mean. The same policy conducted in revenue-neutral form, for example by redistributing revenues on an equal per-capita basis, is estimated to benefit the majority of households in all but the top income quintile, and even the majority of motorist households overall. These important features of the policy are hidden under analysis at the mean. The gains would result from a relatively small estimated transfer from generally higher income households. This is because of the high concentration of estimated motoring emissions and their strong association with income.

Our estimation of who stands to lose and gain financially raises a key question for comparison of ‘tax and dividend’ and ‘cap and share’ variants of the revenue-neutral policy. The marginal propensity to consume varies inversely with income. For example, using Italian data, Japelli and Pistaferri [48] report that the poorest households sampled spend on average c.70% of additional income, whilst the richest spend only c.35%, and estimate that transferring 1% of national income from the richest to the poorest income decile would increase consumption expenditure by 0.33%, Therefore, it cannot be ruled out *a priori* that fuel consumption would increase under tax and dividend, contrary to the environmental goal, despite a higher fuel price. To address that issue requires going beyond static microsimulation. Either policy would plausibly increase consumption expenditure overall.

A limitation of the method presented is that one needs to know whether a unit records a zero value because of a short observation window or for some other reason. In the NTS one can distinguish between infrequency of purchase and non-consumption of motor fuels, because vehicle ownership is recorded. In other consumption surveys, including the widely-used Living Costs and Food Survey, this is not known for many items. So for a long time econometricians have endeavoured to distinguish between non-consumption of meat, tobacco and alcohol, for example, and infrequency of purchase. An implication of the present study is that inclusion of a question to identify non-consumption of important items has potentially large benefits at relatively small cost. Since this may allow researchers to apply simple matching methods to recover the distribution of consumption rates.

## Supporting information

S1 File Supporting Information. (DOCX)
