# Supplementary material for: Something from nothing: Estimating consumption rates using propensity scores, with application to emissions reduction policies
Source: PLoS One. 2017 Oct 11;12(10):e0185538. doi: 10.1371/journal.pone.0185538 (PMC5636092; doi:10.1371/journal.pone.0185538)
Supplement: S1 File — (DOCX) [file pone.0185538.s001.docx]

**Supporting Information**

**
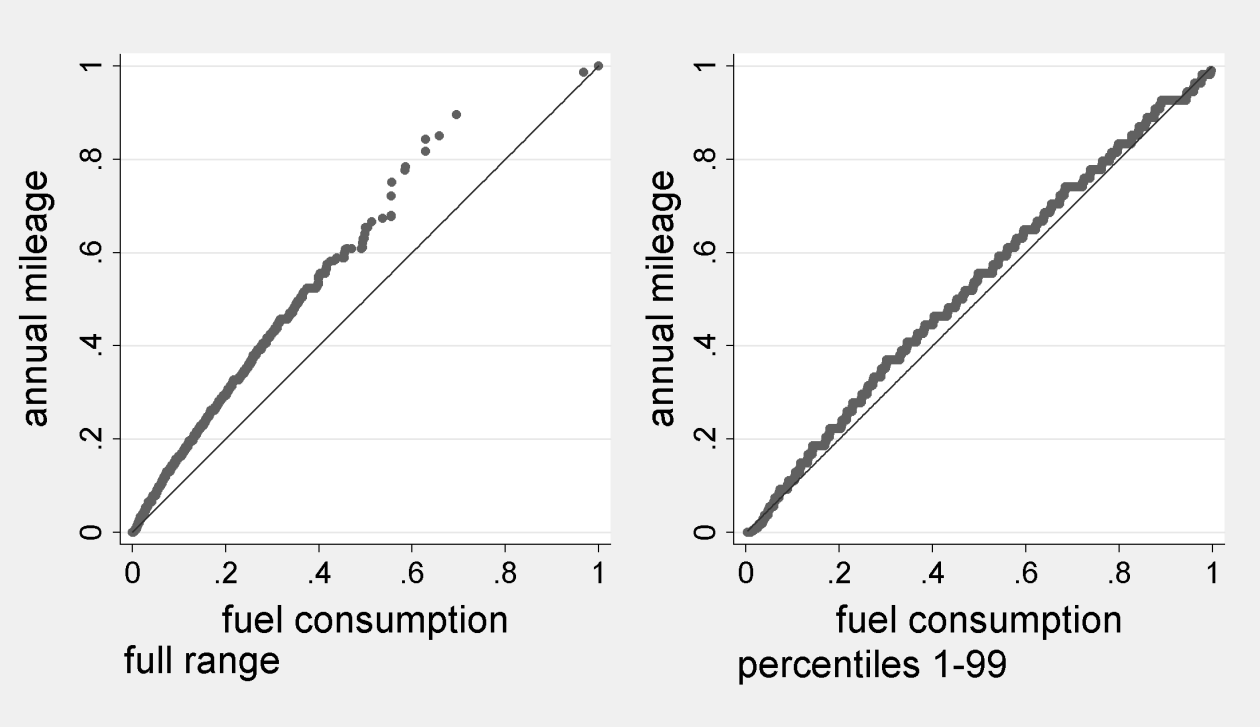
**

**Fig A. Quantile-Quantile plots of estimated fuel purchases against annual mileage; model 1 estimates.**

**
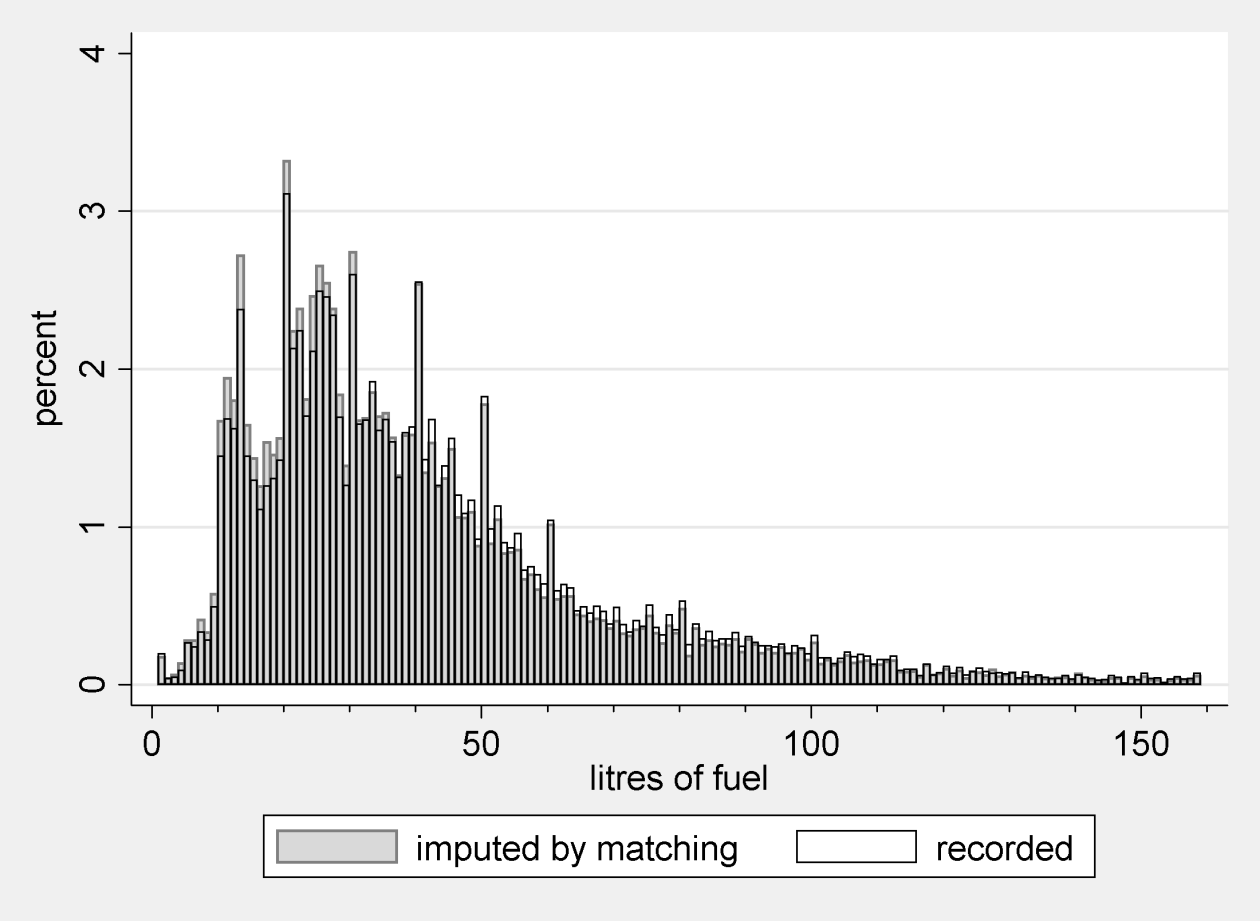
**

**Fig B: quantities at the pump (litres) derived from PSM using model 1**

**
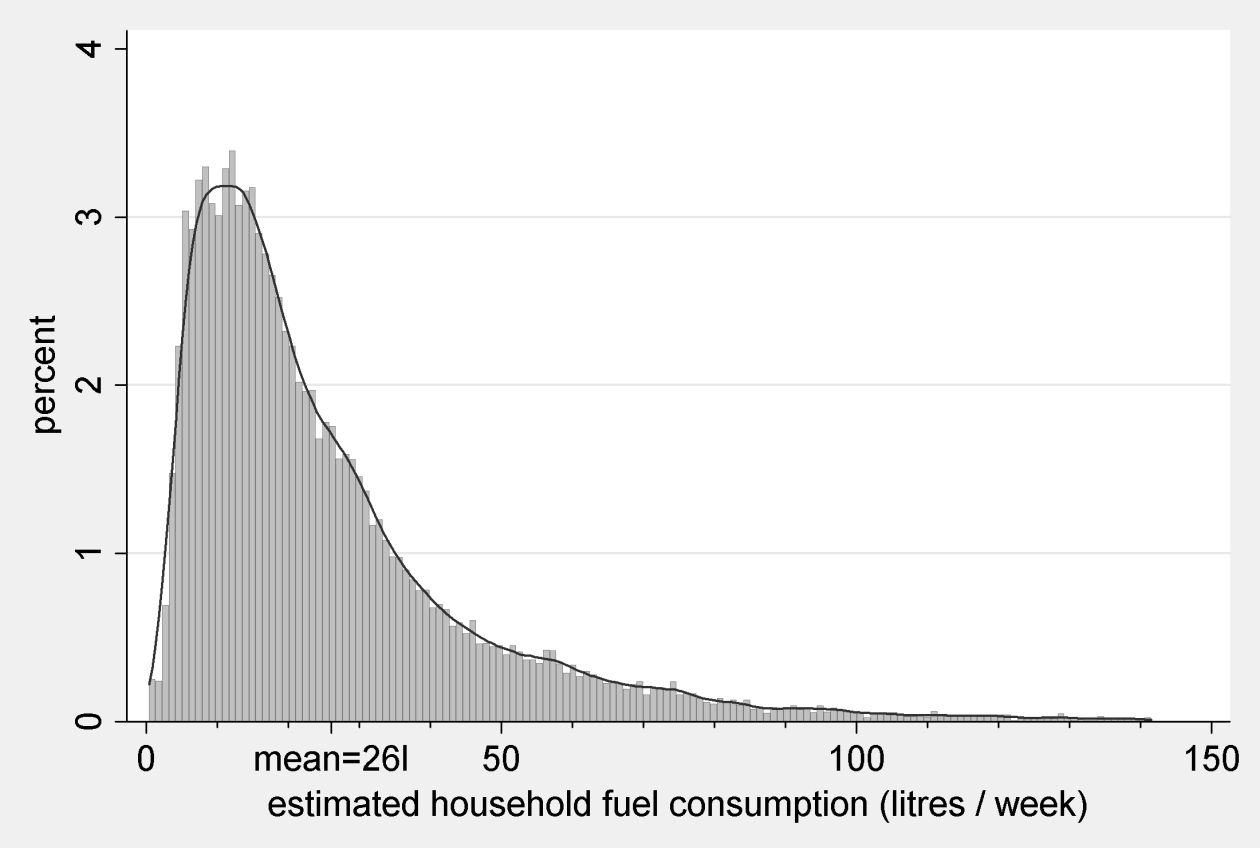
**

**Fig C: Estimates of fuel consumption rates, derived from model 1**

**
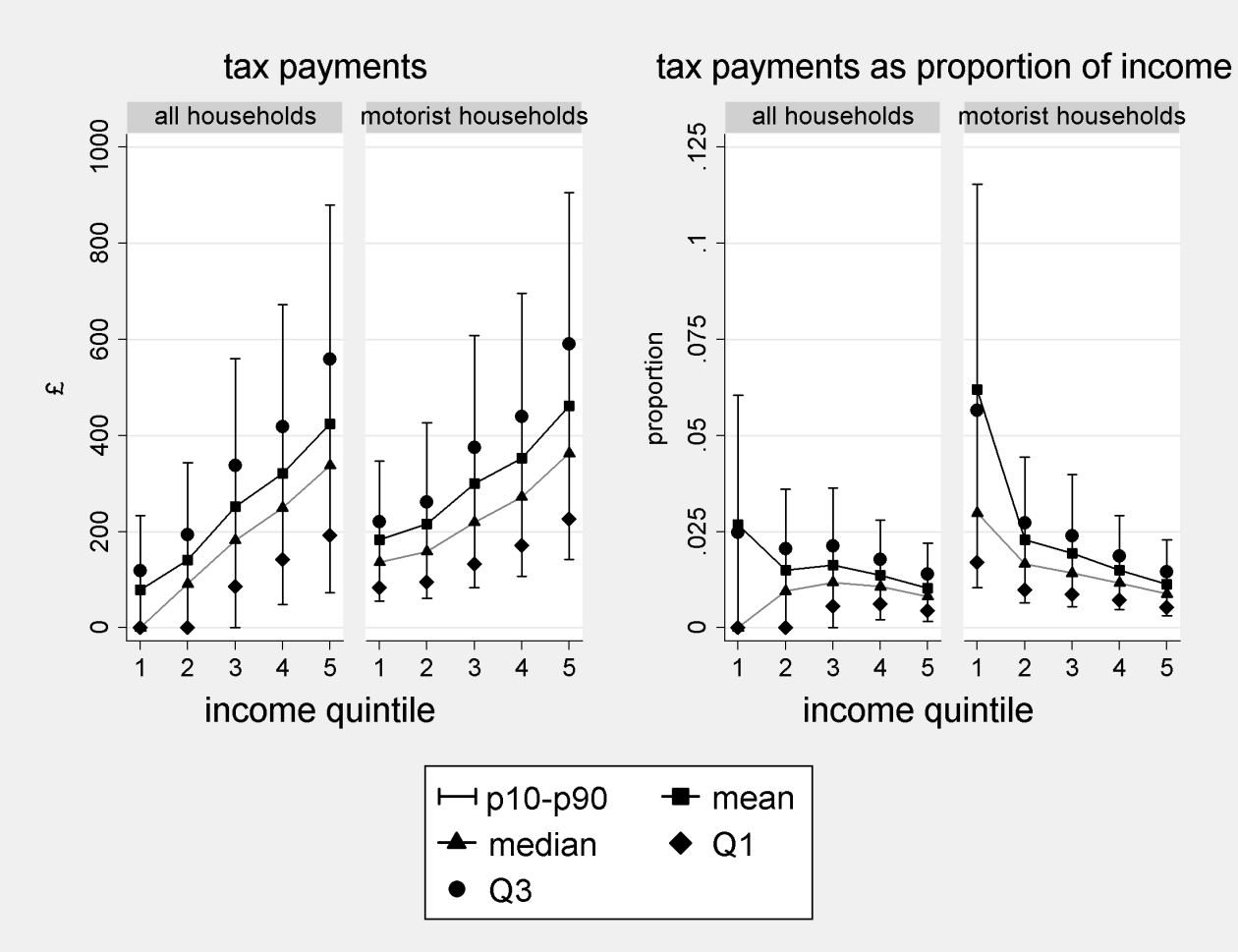
Fig D. Estimated monetary effects of a carbon tax or ration at £100/tCO_2_; model 1 ps**

Note: ‘diary sample’ weights applied, recalculated for the pooled sample.

**
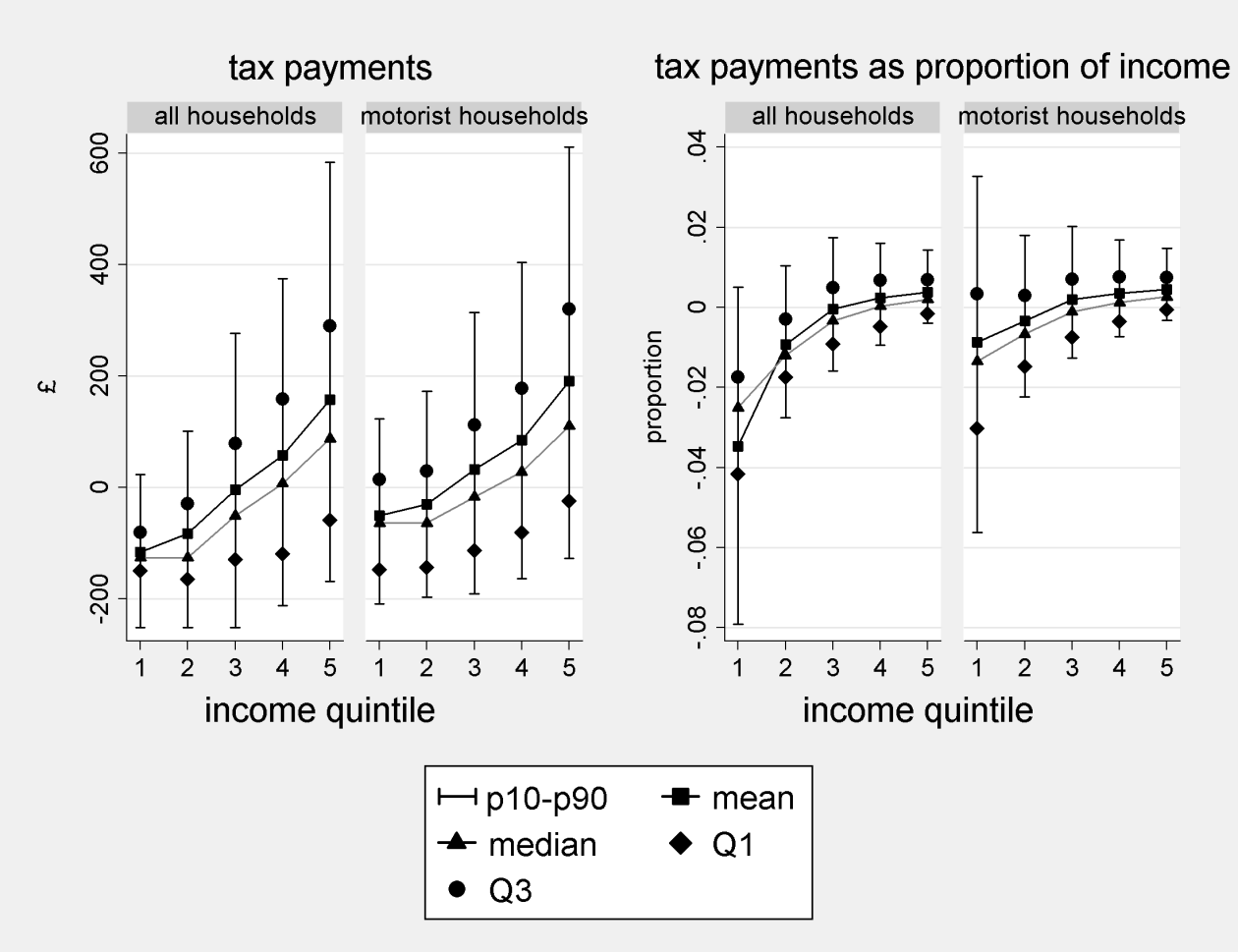
Fig E. Estimated monetary effects of ‘cap and share’ or ‘tax and dividend’ at 100/tCO_2_; model 1 ps**

Note: ‘diary sample’ weights applied, recalculated for the pooled sample.

**Table A. Comparison of the frequency distribution of matches between PSM on the original sample and a bootstrap sample, with e~N(0, 1/30625)**

| Match: | Original sample | | | Bootstrap sample | | |  |
| --- | --- | --- | --- | --- | --- | --- | --- |
| Times used | Freq. | Percent | Cum. | Freq. | Percent | Cum. |  |
| 1 | 6,239 | 61.32 | 61.32 | 6,102 | 60.39 | 60.39 |  |
| 2 | 2,266 | 22.27 | 83.59 | 2,323 | 22.99 | 83.37 |  |
| 3 | 901 | 8.86 | 92.44 | 899 | 8.9 | 92.27 |  |
| 4 | 373 | 3.67 | 96.11 | 380 | 3.76 | 96.03 |  |
| 5 | 173 | 1.7 | 97.81 | 206 | 2.04 | 98.07 |  |
| 6 | 97 | 0.95 | 98.76 | 80 | 0.79 | 98.86 |  |
| 7 | 46 | 0.45 | 99.21 | 44 | 0.44 | 99.3 |  |
| 8 | 32 | 0.31 | 99.53 | 32 | 0.32 | 99.61 |  |
| 9 | 25 | 0.25 | 99.77 | 14 | 0.14 | 99.75 |  |
| 10 | 7 | 0.07 | 99.84 | 7 | 0.07 | 99.82 |  |
| 11 | 4 | 0.04 | 99.88 | 7 | 0.07 | 99.89 |  |
| 12 | 8 | 0.08 | 99.96 | 6 | 0.06 | 99.95 |  |
| 13 | 2 | 0.02 | 99.98 | 4 | 0.04 | 99.99 |  |
| 14 | 2 | 0.02 | 100 | 0 | 0 | 99.99 |  |
| 15 | 0 | 0 | 100 | 1 | .01 | 100 |  |

**Table B. Monte Carlo (MC) simulation results**

| Statistic | Mean of Monte Carlo samples {A} | SD of Monte Carlo samples {A} | SD of bootstrap samples | Mean of Monte Carlo samples {B} | SD of Monte Carlo samples {B} | SD of bootstrap  samples |
| --- | --- | --- | --- | --- | --- | --- |
|  | IP, PSM | IP, PSM | e, IP, PSM |  |  |  |
| p1 | 1.47 | 0.048 | 0.046 | 1.53 | 0.028 | 0.028 |
| p10 | 4.23 | 0.066 | 0.063 | 4.37 | 0.035 | 0.036 |
| p25 | 7.82 | 0.099 | 0.096 | 8.02 | 0.050 | 0.053 |
| p50 | 15.51 | 0.187 | 0.179 | 15.75 | 0.094 | 0.095 |
| p75 | 30.75 | 0.378 | 0.379 | 30.91 | 0.199 | 0.204 |
| p90  p99  mean  sd  skewness  N (reps) | 56.96  164.89  25.97  35.03  6.37  1000 | 0.882  5.193  0.311  2.200  3.110  1000 | 0.856  5.259  0.315  1.417  1.117  1000 per MC sample | 56.75  161.20  25.97  34.04  6.11  1000 | 0.467  2.918  0.162  0.897  1.833  1000 | 0.470  2.940  0.165  0.780  0.788  1000 per MC sample |

Notes

1. In each MC simulation sample we draw 42,700 observations *g* from a lognormal distribution selected to roughly resemble our model 2 estimates: *g~*15.75.e^(N[0,1])^
2. In columns 1-3, each MC sample in sample set {A} is modified to mimic infrequent purchase (IP), replacing *g* with zeros according to an indicator variable (I), and inflating non-zero z values accordingly. p(I=1) depends on one covariate included in the simulated dataset. We estimate p(I=1) and conduct PSM to recover *g*. For each MC sample we also draw bootstrap samples. For each bootstrap sample we estimate p(I=1), add *e,* conduct PSM and multiply values by $\hat{ps}$, as in the main text.
3. In columns 4-6 for each MC sample in sample set {B} we simply draw bootstrap samples. Comparing columns 1-3 and 4-6, it appears that the underestimated bootstrap standard errors for sd and skewness in column 3 are at least partly associated with skewness in the underlying distribution.
